# A117 EXPECT THE UNEXPECTED. UNUSUAL PATHOLOGY FINDINGS FROM ROUTINE POLYPECTOMY. A REVIEW OF SUBMUCOSAL GI LESIONS

**DOI:** 10.1093/jcag/gwae059.117

**Published:** 2025-02-10

**Authors:** O Adesina

**Affiliations:** Department of Medicine, University of Saskatchewan, Saskatoon, SK, Canada

## Abstract

**Background:**

Granular cell tumours (GCTs) are rare benign submucosal neoplasms. They most commonly arise from neural or Schwann cells. While GCTs are frequently encountered in the oral cavity, skin, and breast, gastrointestinal involvement remains a clinical rarity. This case series highlights the incidental detection of GCTs during routine colonoscopy. In this setting, we provide insights into the diagnostic challenges and management approaches for such submucosal lesions.

**Aims:**

To present a case series of two patients with incidentally discovered GCTs during routine colonoscopy, discuss the diagnostic and management strategies for GCTs, and offer a review of current literature with implications for clinical practice.

**Methods:**

We present two cases of GCTs identified during routine colonoscopic polypectomy. The clinical presentations, diagnostic processes, and management strategies were analyzed using the existing literature. Particular focus was given to the challenges of identifying submucosal lesions in the gastrointestinal tract and determining appropriate follow-up care based on histopathological findings.

**Results:**

The first case is a 75-year-old man who underwent a colonoscopy after a positive fecal immunochemical test (FIT), with normal physical and laboratory findings. Multiple polyps were identified, and histopathology confirmed a GCT in the cecum. The patient was asymptomatic, and a surveillance colonoscopy was scheduled.

The second case is a 74-year-old woman who was referred to gastroenterology after a positive FIT. She was asymptomatic, with normal physical and laboratory findings. Initial colonoscopy revealed two resected polyps. A follow-up colonoscopy a year later identified three additional polyps, one of which was a GCT. Surveillance colonoscopy was advised in four years.

GCTs of the gastrointestinal tract are rare, most commonly found in the distal esophagus (about 60%). Typically submucosal, they rarely invade the mucosa or muscularis propria. Other notable subepithelial lesions include gastrointestinal stromal tumours (GISTs), leiomyomas, lipomas, ectopic pancreatic tissue, and neuroendocrine tumours. Unlike GCTs, GISTs and leiomyosarcomas are more often malignant. Endoscopic, radiologic, and endosonographic assessments assist in the risk stratification of subepithelial lesions, with one study demonstrating these features having a 92.9% specificity for detecting GISTs. Histology remains crucial for confirmation of diagnosis. Management of benign GCTs may involve endoscopic resection or surveillance, whereas malignant cases may require more extensive treatment, including adjuvant chemotherapy or radiotherapy.

**Conclusions:**

GCTs are rare findings in gastrointestinal pathology. Treatment involves resection or close surveillance. Further research is needed to refine optimal diagnostic and therapeutic approaches for these uncommon neoplasms.

**Funding Agencies:**

None

